# Understanding Antidiabetic Potential of Oligosaccharides from Red Alga Dulse *Devaleraea inkyuleei* Xylan by Investigating α-Amylase and α-Glucosidase Inhibition

**DOI:** 10.3390/molecules29071536

**Published:** 2024-03-29

**Authors:** Martin Alain Mune Mune, Tadashi Hatanaka, Hideki Kishimura, Yuya Kumagai

**Affiliations:** 1 Faculty of Science, University of Maroua, Maroua P.O. Box 814, Cameroon; 2 Okayama Prefectural Technology Center for Agriculture, Forestry and Fisheries, Research Institute for Biological Sciences (RIBS), Okayama, 7549-1 Kibichuo-cho, Kaga-gun, Okayama 716-1241, Japan; hatanaka@bio-ribs.com; 3 Laboratory of Marine Chemical Resource Development, Faculty of Fisheries Sciences, Hokkaido University, Hakodate 041-8611, Japan; i-dulse@fish.hokudai.ac.jp

**Keywords:** dulse, xylan, oligosaccharides, α-glucosidase inhibition, maltase-glucoamylase, α-amylase inhibition, enzyme kinetics, molecular docking

## Abstract

In this study, the α-glucosidase (maltase-glucoamylase: MGAM) and α-amylase inhibitory properties elicited by xylooligosaccharides (XOSs) prepared from dulse xylan were analysed as a potential mechanism to control postprandial hyperglycaemia for type-2 diabetes prevention and treatment. Xylan was purified from red alga dulse powder and used for enzymatic hydrolysis using Sucrase X to produce XOSs. Fractionation of XOSs produced xylobiose (X2), β-(1→3)-xylosyl xylobiose (DX3), xylotriose (X3), β-(1→3)-xylosyl-xylotriose (DX4), and a dulse XOS mixture with *n* ≥ 4 xylose units (DXM). The different fractions exhibited moderate MGAM (IC_50_ = 11.41–23.44 mg/mL) and α-amylase (IC_50_ = 18.07–53.04 mg/mL) inhibitory activity, which was lower than that of acarbose. Kinetics studies revealed that XOSs bound to the active site of carbohydrate digestive enzymes, limiting access to the substrate by competitive inhibition. A molecular docking analysis of XOSs with MGAM and α-amylase clearly showed moderate strength of interactions, both hydrogen bonds and non-bonded contacts, at the active site of the enzymes. Overall, XOSs from dulse could prevent postprandial hyperglycaemia as functional food by a usual and continuous consumption.

## 1. Introduction

Dulse (*Devaleraea inkyuleei*, formerly *Palmaria palmata* in Japan) is a popular red alga widely distributed around the North Pacific and North Atlantic. It is primarily used for food due to its high nutritional potential, including high protein, mineral and vitamin content, as well as a high fibre and polyphenol content [1]. Red algae also contain bioactive compounds, and it has been shown that long-term consumption of red algae is linked to the prevention of diseases such as diabetes, cancer, obesity, and inflammation-relation related complications. The prevention of type-2 diabetes (T2D) is particularly important because this disease is a public health problem worldwide, with a high percentage of mortality among adults aged 20–79 years old (14.5%), and it affects 1 in 10 adults. Moreover, healthcare costs for the treatment of T2D are high and the utilization of actual medicines based on synthetic compounds presents many side effects [2]. Therefore, the complementary approach using food or food constituents could be used as a safe and low-cost alternative for the management of T2D, although these constituents are not as potent as synthetic drugs. Investigation of the antidiabetic potential of dulse constituents could then be promising.

Dulse is rich in protein and carbohydrates, with their contents depending on seasonal and nutritional conditions. Peptides derived from dulse protein exhibited a suppression effect on hypertension [3]. An antioxidant effect was found from the peptides and low molecular weight amino acid-like compounds [4,5,6]. The protein sequence of the major proteins has also been revealed by genome sequencing [7]. Proteins from dulse also contain encrypted peptides with dipeptidyl peptidase-IV (DPP-IV) inhibitory activity in their sequences, and hydrolysis with corolase PP released peptides with an IC_50_ range within 43–159 µM [8]. DPP-IV is a target enzyme for the treatment of T2D. Carbohydrates in dulse mainly consist of cell-wall polysaccharides such as cellulose, agar, galactan or carrageenan, and xylan is the major one. Dulse xylan (DX) is a water-soluble linear polymer mainly composed of β-(1→3) and β-(1→4)-linked D-xylopyranose units, with one β-(1→3) linkage for every β-(1→4) xylotetraose [9]. Xylan is partially degraded in the gut by microorganisms to produce xylose and XOSs. DX was also successfully used for the in vitro production of XOSs using commercial enzymes Hemicellulase amano 90 and Sucrase X [10]. The growth of *Bifidobacterium adolescentis* by DX3 was confirmed [11]. In addition, we found an effective method for the production of oligosaccharides from DX with a high percent of β-(1→3) linkages using recombinant enzymes from *Streptomyces thermogriseus* [12]. XOSs are not hydrolysed by gastrointestinal enzymes or absorbed in the intestine but stimulate a number of beneficial biological functions for humans. It has been demonstrated that XOSs promoted *Bifidobacteria* growth and provided short-chain fatty acids, which are absorbed by the colonic mucosa and decrease the plasma free fatty acid level [13,14].

It has also been shown that XOS supplementation in T2D patient diet reduced plasma glucose concentration and prevented diabetes complications [15]. Basically, XOSs exerted antidiabetic activity through multiple mechanisms, including affecting glucose uptake (inhibition of carbohydrates hydrolysing enzymes such as α-glucosidase and α-amylase, inhibition of intestinal glucose transport, inhibition of hepatic glucose output, and the delay of intestinal absorption of carbohydrates), affecting the incretin effect (stimulation of glucagon-like peptide-1 and the inhibition of DPP-IV), and mimicking the insulin action (insulin release or sensitivity and alleviation of oxidative stress) [16,17].

However, the inhibition of α-glucosidase and α-amylase is one of the effective therapeutic approaches to decrease postprandial hyperglycaemia as well as reduce the onset of diabetic complications. A study on neoagarooligosaccharides showed little inhibition of α-amylase and significant inhibition of α-glucosidase, particularly for the neoagarotetrose and neoagarohexose mixture [18]. In addition, a study on polysaccharides from wheat bran showed that xylan with substitutions at O-3 positions by different sugars exhibited a strong inhibitory effect on the activity of α-glucosidase (maltase-glucoamylase: MGAM) and α-amylase [19]. Therefore, it could be expected that XOSs from DX, particularly those with mixed β-(1→3)/β-(1→4) linkages, could exert an inhibitory effect on starch digestive enzymes. The aim of this study was to investigate the inhibitory potential of XOSs prepared from DX, as a mechanism to explain their antidiabetic activity.

## 2. Results and Discussion

### 2.1. Production and Purification of Xylooligosaccharides from Dulse Xylan

The production of XOSs by enzymatic hydrolysis of xylan usually requires xylan extraction to minimize interference during enzyme action. In this regard, dulse powder contained other compounds, such as proteins, pigments, or other carbohydrates. These components are tightly associated with xylan in the cell wall and extracted with xylan during the extraction procedure since these share many similar chemical properties with xylan. It is well-known that the bioactivities of oligosaccharides are closely correlated to their molecular structure and composition [20]. Understanding these aspects of dulse xylan XOSs are essential for their application as functional foods.

**Figure 1:**
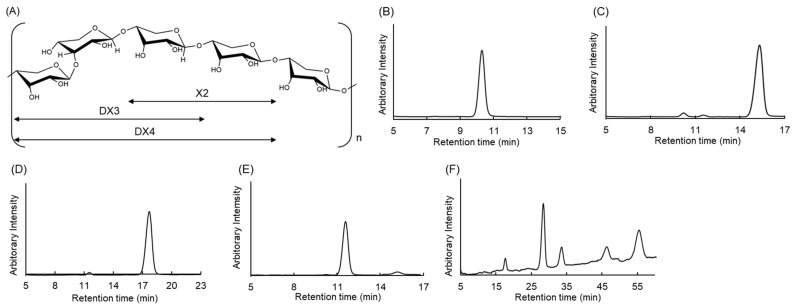
Chemical structure of DX and purity of XOSs. (**A**), DX; (**B**–**F**), HPLC profile for the purified X2; (**B**), X3; (**C**), DX3; (**D**), DX4; and (**E**), DXM (Dulse XOSs with the number of monomers ≥ 4, DXM) (**F**).

We developed the extraction process from dulse powder, resulting in a dulse xylan-rich fraction instead of pure xylan [10,21]. In this study, xylan was extracted from dulse powder with a yield of 29%. DX was then hydrolysed by Sucrase X, an enzyme producing a high yield of XOSs with the composition of xylose (15%), X2 (21%), X3 (6%), DX3 (21%), and DX4 (17%). Enzymatically produced dulse-specific xylo-oligosaccharides contained β-(1→3)-linkages at the non-reducing ends [10]. XOSs were separated by an activated carbon adsorption, and the products were analysed by TLC and HPLC (Figure 1 and Figure S1, Table S1).

It is observed that β-(1→4)-xylobiose (X2) was fractionated using 5% (*v*/*v*) ethanol, with a retention time of 10.258 min by HPLC (Figure 1A,B), and β-(1→4) xylotriose (X3) using 10% (*v*/*v*) ethanol (retention time of 15.274 min by HPLC) (Figure 1C). The fraction obtained using 15% (*v*/*v*) ethanol with a retention time of 11.583 min by HPLC was previously identified as β-(1→3)-xylosyl-β-(1→4)-xylobiose (DX3) (Figure 1A,D), and the fraction at 20% (*v*/*v*) ethanol with a retention time of 17.700 min by HPLC was identified as β-(1→3)-xylosyl-β-(1→4)-xylotriose (DX4) (Figure 1A,E). This result is based on the previous study of evaluating Sucrase X activity toward β-(1→3)-glycosidic bond in dulse xylan [10]. A mixture of XOSs from dulse (DXM) with the number of xylose units ≥4 was also collected at 50% (*v*/*v*) ethanol (Figure 1F). Afterwards, fractions X2, X3, DX3, DX4, and DXM were analysed for MGAM and α-amylase inhibition for better understanding of the antidiabetic potential of dulse xylan. It should provide a concise and precise description of the experimental results, their interpretation, as well as the experimental conclusions that can be drawn.

### 2.2. Dulse XOS Inhibitory Activity against MGAM and α-Amylase

Inhibition of α-glucosidase and α-amylase is an effective therapeutic approach to decrease postprandial hyperglycaemia and, consequently, for the treatment and prevention of T2D [16]. In this regard, starch and glycogen are mainly converted to maltose by pancreatic and salivary α-amylase and then to glucose by α-glucosidase at the brush border of the small intestine. Then, glucose is absorbed and transported in the bloodstream.

**Figure 2:**
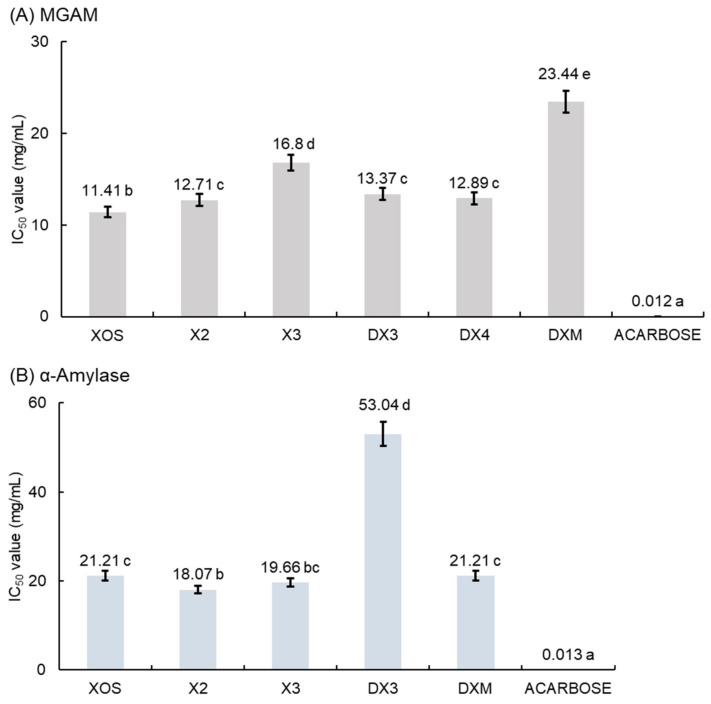
Half-maximum inhibitory concentration (IC_50_) values for the inhibition of XOSs and acarbose. Inhibition of XOSs toward MGAM (**A**) and α-amylase (**B**) (DX4 produced 2.9% inhibition at 10.64 mg/mL). Means with different letters are significantly different (*p* < 0.05). Comparison was performed using a one-way ANOVA with a Duncan post hoc test.

The IC_50_ of the different XOSs and acarbose for the inhibition of MGAM and α-amylase are presented in Figure 2. It was observed that DX hydrolysate exhibited a significantly (*p* < 0.05) lower IC_50_ compared to XOS. A comparable IC_50_ (12.71–13.37 mg/mL) was also found for X2, DX3, and DX4, while X3 and DXM presented a significantly higher IC_50_ (16.80 mg/mL and 23.44 mg/mL, respectively). These results showed that for XOSs with n ≥ 3, the presence of a β-(1→3)-glycosidic bond was more suitable for the inhibition of MGAM than a β-(1→4)-glycosidic bond alone. However, the commercial drug acarbose exhibited stronger inhibition (IC_50_: 0.012 mg/mL) compared to XOS and DX hydrolysate. The higher inhibition observed for DX hydrolysate compared to XOS was probably due to a synergistic action of XOS on the inhibition of MGAM. A similar effect was observed with bioactive compounds from finger millet [22]. Similar results were also obtained for neoagarooligisaccharides at 10 mg/mL; in particular, neoagarobiose, neoagarotetraose, and neoagarohexaose showed inhibition rates of 35.3%, 33.2%, and 17.0%, respectively. The strongest inhibition was observed for the neoagarooligosaccharide mixture (42.3% inhibition at 10 mg/mL) [23].

For α-amylase inhibition, the lower IC_50_ was noticed for X2 (IC_50_: 18.07 mg/mL) followed by X3 (IC_50_: 19.66 mg/mL); then, DXM and DX hydrolysate produced the same inhibition (IC_50_: 21.21 mg/mL). A low inhibition of DX3 on α-amylase was noticed (IC_50_: 53.04 mg/mL) and DX4 produced very weak inhibition (2.9% inhibition at 10.64 mg/mL) (Figure 2B). Neoagarooligosaccharides also presented very low inhibition to α-amylase (≤15% inhibition at 1 mg/mL) [23]. Iminosugars also inhibit glycanase activities [24]. Iminosugars (1.41 μM) and phenolic compounds (9–86 μM) are known to exhibit stronger inhibition of MGAM [25,26]. Similarly, iminosugars (0.45–2.0 μM) and phenolic compounds (0.498 mg/mL) are also known to exhibit stronger inhibition of α-amylase [27,28]. While the activity of oligosaccharides derived from natural plants is weak, their intake is anticipated to provide functionality due to their abundant presence in common foods.

### 2.3. Kinetics Studies

Although the MGAM inhibitory activity of XOS was lower than that of the common drug acarbose, their utilization to control postprandial hyperglycaemia present superior safety and a reduced or low risk of adverse effects. Therefore, investigation on enzyme kinetics becomes crucial to understand the molecular mechanisms involved in the interaction between XOS and carbohydrate digestive enzymes. In addition, it could be assumed that XOSs could be used as a structural basis for the development of further functional foods. This is because chemical modifications of oligosaccharides, such as acylation, sulfation, and phosphorylation, have the potential to improve the inhibition of carbohydrate digestive enzymes [29]. Therefore, Michaelis–Menten and Lineweaver–Burk kinetics were applied to study the inhibition of XOSs from dulse to α-amylase and MGAM.

#### 2.3.1. Kinetics of the Inhibition of MGAM by XOSs

**Figure 3:**
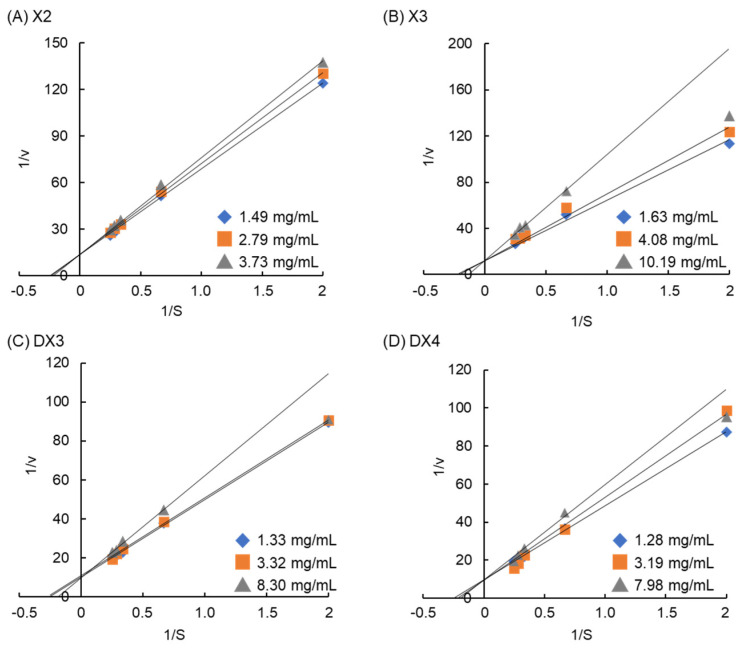
Lineweaver–Burk plot for the inhibition of MGAM by XOSs at different concentrations of maltose. (**A**), X2; (**B**), X3; (**C**), DX3; and (**D**), DX4. Lineweaver–Burk plots are generated, excluding data points associated with a maltose concentration of 4 (1/S) for 10.19 mg/mL of X3, 8.30 mg/mL of DX3, and 7.98 mg/mL for DX4.

The inhibition of MGAM by XOSs (X2, X3, DX3, and DX4) from dulse was exhibited by Michaelis–Menten and Lineweaver–Burk plots (Table 1, Figure 3 and Figure S2). Results from the Michaelis–Menten plot clearly demonstrated that XOSs inhibited MGAM in a concentration-dependent manner, increasing inhibition with the inhibitor concentration. The Lineweaver–Burk plot trend was similar for the four XOSs, with the graphs intercepted on the y-axis and the value of 1/*V*_max_ remaining constant, while the slope of the graph increased with the XOS concentration. These results confirmed that X2, X3, DX3, and DX4 act as competitive inhibitors to MGAM (Table 1). No significant difference (*p* > 0.05) in *V*_max_^app^ was observed in the presence and the absence of inhibitor. In addition, the unchanged *V*_max_^app^ observed with increasing XOS concentration suggested that the inhibition was not irreversible. The *K*_i_ for XOSs were almost similar and in the range of 15.20–23.33 mg/mL (Table 1), but much higher than the *K*_i_ of acarbose (62 µmol/L, 0.04 mg/mL) for MGAM, as reported [30].

XOSs could not lead to an accumulation effect in vitro, confirming their safety for human health [31]. XOSs from dulse probably bound reversibly to or near the active site of MGAM through non-covalent bonds, forming stable complexes that prevent the binding of maltose to the enzyme. It could be observed that *K*_m_^app^ (apparent Michaelis–Menten constant) increased with the inhibitor concentration, while *V*_max_^app^ (apparent maximum reaction rate) was almost the same (Table 1). This means that XOS blocked maltose from accessing the active site of the enzyme. Inhibition of MGAM by XOSs from finger millet bran was also found to be competitive [22], while the inhibition by arabinoxylan oligosaccharides to MGAM was found to be non-competitive [32].

#### 2.3.2. Kinetics of the Inhibition of α-Amylase by XOSs

**Figure 4:**
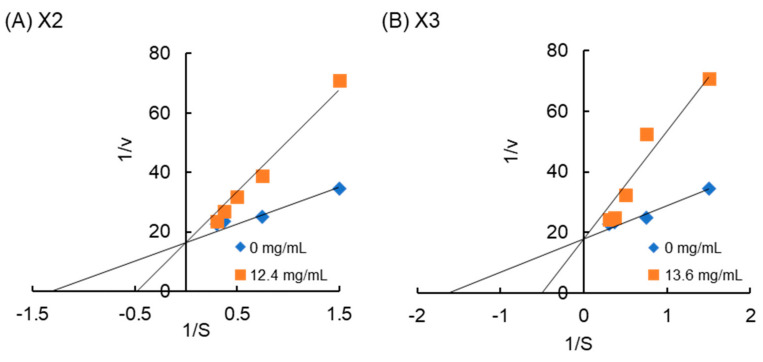
Lineweaver–Burk plot for the inhibition of α-amylase by XOSs at different concentrations of starch: (**A**) X2 and (**B**) X3. The equations are as follows: X2 (0 mg/mL): y = 12.5x + 16.5 (R^2^ = 0.96); X2 (12.4 mg/mL): y = 34.1x + 16.5 (R^2^ = 0.99); X3 (0 mg/mL): y = 10.9x + 18.0 (R^2^ = 0.96); and X3 (13.6 mg/mL): y = 35.4x + 18.0 (R^2^ = 0.94).

XOS, X2, and X3 exhibited stronger inhibition on α-amylase (Figure 4 and Figure S3). Results from the Michaelis–Menten graph clearly showed that X2 and X3 decreased the initial velocity of the enzyme. The inhibition effect was less observed at high starch concentrations. This result was also shown in the Lineweaver–Burk graph, where the plots in the presence and absence of the inhibitor intercepted on the y-axis, while the value of 1/*V*_max_ remained constant (Table 2).

This result concluded that X2 and X3 act as competitive inhibitors to α-amylase, binding to the active site of α-amylase. On the other hand, the inhibition of α-amylase by the XOS mixture from arabinoxylan finger millet [22] affected not only *K*_m_ but also *V*_max_. Similar results were found using xylan substituted at O-3 positions by arabinose from wheat bran [19]. It is concluded that XOS and xylan moderately inhibit α-amylase activity.

### 2.4. Prediction of the Interaction between Digestive Enzymes and XOSs

It has been shown that XOSs from dulse xylan inhibited α-amylase and MGAM by reversible binding at the active site, then preventing the substrate from the active site of the enzyme. Investigation on the molecular interaction between XOSs and carbohydrate digestive enzymes was then performed at the atomic level using molecular docking. Docking was also performed using acarbose, a well-known inhibitor of α-amylase and MGAM, to validate and compare the docking results. In addition, the root-mean-square deviation (RMSD) after docking XOSs and acarbose was less than 1 Å, confirming the validity of docking procedure. Results were expressed in terms of affinity energy, amino acid residues interacting, and the number of hydrogen bond as well as non-bonded contacts (Table 3). Generally, hydrogen bonds are directly involved in the specific residues at the active site of the enzyme to specific groups on the inhibitor. Non-bonding contacts are mostly involved in the formation of a stable enzyme–inhibitor complex [33]. Interactions between enzymes and XOSs were finally visualized using 2D graphs.

#### 2.4.1. Interaction between MGAM and XOSs

**Figure 5:**
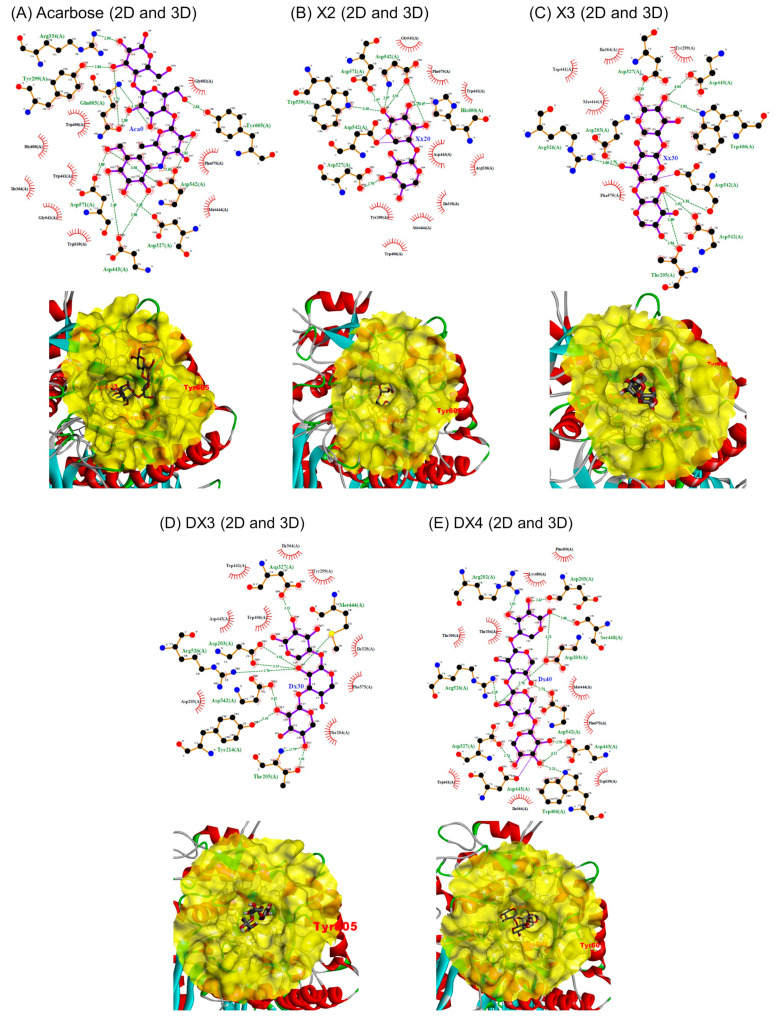
Two-dimensional and three-dimensional views of the interaction between human MGAM and XOSs ((**A**), Acarbose; (**B**), X2; (**C**), X3; (**D**), DX3; and (**E**), DX4).

It has been reported that the active site of MGAM forms a pocket primarily composed of C-terminal β-strand residues within the (β/α)_8_ barrel structure, and this site includes the −1 and +1 subsite of sugars [34]. As expected, acarbose effectively bound to the active site, primarily occupying the subsites −1 and +1, with the first two rings of the non-reducing end (the acarviosine unit) (Figure 5). This interaction was maintained through hydrogen bonds with amino acid residues Tyr299, Asp443, and Asp542 at the subsite −1. Additionally, there was interaction at the subsite +1 through hydrogen bonds with Asp542, along with non-bonded interactions with Trp406, Asp542, and Tyr605 [34]. Notably, Asp542 is a well-conserved amino acid within glycosyl hydrolase family 31 and plays a critical role in the nucleophilic double displacement reaction, in association with Asp443, leading to the hydrolysis of the α-glucosidic linkage at the non-reducing end of the oligosaccharide.

Acarbose exhibited a stronger affinity energy (−7.4 kcal/mol) compared to XOSs, forming 13 hydrogen bonds and 88 non-bonded contacts with the enzyme. However, for XOSs, the affinity energy was strongest for DX4, followed by X2, X3, and DX3, and the number of hydrogen bonds with the enzyme followed the order DX4 > DX3 = X3 > X2. The strength of interaction between XOSs and the amino acid residues at the subsites −1 and +1 within the enzyme’s active site was also crucial for inhibition, as the distance of hydrogen bonds was generally ≥3 Å for X3. Furthermore, non-bonded interactions at the active site between XOSs and MGAM likely increased the strength of the interaction.

X3 exhibited a lower number of non-bonded contacts (63) with the enzyme, while X2 had a higher number of contacts (96), followed by DX4 (79) (Table 3 and Figure 5B–E). There were a substantial number of non-bonded contacts between X2 and MGAM, particularly between X2 and almost all amino acid residues at the subsite −1, as well as with Phe405, Trp406, Arg526, and Tyr605 at subsite +1. The oligosaccharides investigated here had regions capable of binding to both subsite −1 and +1. From the IC_50_ values, X2 exhibited strong inhibition, while in silico analysis suggested stronger binding affinities for others. Differences in these values were expected to decrease due to improper binding for the subsite of the enzyme. These findings provide a general explanation for the in vitro inhibition of MGAM by XOSs derived from DX.

#### 2.4.2. Interaction between α-Amylase and XOSs

**Figure 6:**
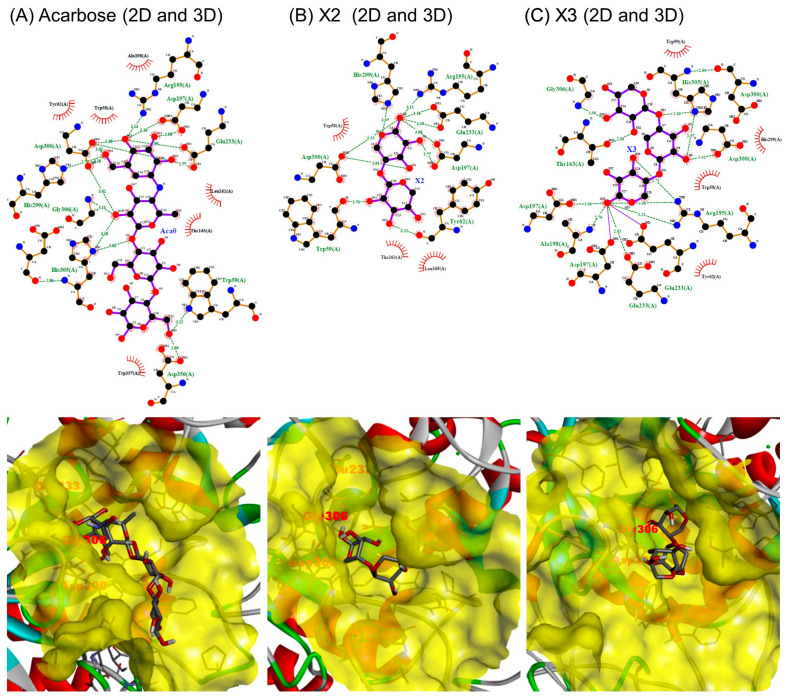
Two-dimensional and three-dimensional views of the interaction between α-amylase and XOSs ((**A**), Acarbose; (**B**), X2; and (**C**), X3).

Acarbose bound to the active site of α-amylase through the formation of 16 hydrogen bonds and 69 non-bonded contacts, with an affinity energy of −5.1 kcal/mol. Most of the hydrogen bond interactions occurred through the first two rings of the non-reducing end, which constitute the acarviosine unit. These rings occupied both the −1 subsites (interacting with Arg195, Asp197, His299, and Asp300) and the +1 subsite (interacting with Glu233 and Asp300). Additionally, acarbose engaged with α-amylase at the +2 subsite through non-bonded interactions with Gly306 (Figure 6A and Table 3).

These findings align with similar results observed through crystallographic analysis [35]. Furthermore, Asp197 and Glu233 are notably crucial amino acids in α-amylase because they play a significant role in driving the nucleophilic displacement mechanism during the hydrolytic reaction. Meanwhile, Asp300 is responsible for coordinating a water molecule, which likely plays a key role in the hydrolytic reaction of α-glucosidic linkage [35,36,37].

XOSs from dulse interacted with α-amylase through the formation of 10 and 12 hydrogen bonds and 57 and 63 non-bonded contacts, respectively, for X2 and X3. Specifically, at the subsite −1, X2 interacted with amino acid residues, forming one hydrogen bond with Arg195 (distance of 3.114 Å), two hydrogen bonds with Asp197 (distances of 2.766 and 3.001 Å), one hydrogen bond with His299 (distance of 3.069 Å), and two hydrogen bonds with Asp300 (distances of 2.912 and 3.229 Å).

No hydrogen bonds were observed between X2 and α-amylase at the sugar subsite +1. Similarly, X3 predominantly formed hydrogen bonds with amino acid residues at the subsite −1, with only one hydrogen bond at the subsite +1 (distance of 3.229 Å, as shown in Figure 6B,C, and Table 3). The inhibition by X2 and X3 for α-amylase is similar, suggesting that inhibition occurs at the smallest inhibitory unit, xylobiose, which occupies subsites −1 and +1. The low inhibition by DX3 for α-amylase may be attributed to the difference in the linkage at the non-reducing end, either β-(1→3) or β-(1→4)-linkages. The lower number of hydrogen bonds between XOSs and α-amylase, as well as the weaker interaction at the subsite +1, helps explain the reduced inhibition potential of XOSs from dulse in comparison to acarbose, as observed in in vitro experiments. The effects on rats were demonstrated by the addition of 6% XOS on dietary food [13]. Since dulse consists of carbohydrates up to 40%, this may suggest the necessary intake of other ingredients containing xylan or XOSs, in addition to dulse.

## 3. Materials and Methods

### 3.1. Materials

Samples (*D. inkyuleei*) were collected on the coast of Hakodate, Hokkaido Prefecture, Japan. Sucrase X (from *Trichoderma longibrachiatum* > 25,000 U/g at pH 5.0, 40 °C) was procured from Mitsubishi-Chemical Foods Corporation (Tokyo, Japan). Xylose, β-(1→4)-xylobiose (X2) and β-(1→4)-xylotriose (X3), porcine stomach pepsin (EC 3.4.23.1), and bovine pancreatic trypsin (EC 3.1.21.4) were purchased from FUJIFILM Wako Pure Chemical (Osaka, Japan). β-(1→4)-Xylotetraose (X4) was purchased from Megazyme (Bray, Ireland). A Sugar-D column (4.6 × 250 mm) was purchased from Nacalai Tesque (Kyoto, Japan). α-amylase from porcine pancreas (EC 3.2.1.1), type VI-B (≥5 units/mg solid), was purchased from Sigma Aldrich (St. Louis, MO, USA).

### 3.2. Preparation of Dulse Xylan

Xylan extraction was carried out as previously reported with some modifications [12]. Frozen dulse was lyophilized and homogenized into a fine powder. The powder was suspended in 40-volumes (*w*/*v*) of distilled water, stirred for 60 min and autoclaved at 121 °C for 20 min. The mixture was then subjected to α-amylase treatment (1%, *w*/*w*) at 50 °C overnight, and the enzyme was inactivated by heating at 100 °C for 10 min. The mixture was centrifugated at 15,000× *g* for 10 min at 4 °C and dialyzed against distilled water using a dialysis tube of 3000 Da molecular weight cutoff (EIDIA Co., Ltd., Tokyo, Japan), and freeze-dried. The resulting powder was mixed with distilled water (1/20, *w*/*v*) and the same volume of 2-propanol, stirred for 5 min and allowed to stand for 30 min, then centrifuged at 8000× *g* at 5 °C for 5 min. The extraction procedure was repeated once, and the pellet was dissolved in water. The residual solvent was removed by evaporation, and the solution was freeze-dried. The dried sample was used as DX.

### 3.3. Preparation of XOSs and Characterization

For the hydrolysis by Sucrase X, DX of 10 mg/mL in a 10 mM sodium acetate buffer (pH 6.0) was hydrolysed using 2.0% (*w*/*w*) Sucrase X at 50 °C for 3 h [10]. The enzyme reaction was stopped by heating at 100 °C for 10 min. The mixture was centrifuged at 15,000× *g* at 4 °C for 10 min. The supernatant was freeze-dried, and DX hydrolysate was kept at −30 °C before use. XOSs were dissolved in water and applied to an activated carbon column (φ 3 × 35 cm) pre-equivalated with distilled water. The carbohydrates were eluted by a stepwise system consisting of 0%, 5%, 10%, 15%, 20%, and 50% ethanol at a flow rate of 1 mL/min. Fractions were analysed by thin-layer chromatography (TLC) and HPLC (Table S1).

### 3.4. Thin-Layer Chromatography

The TLC analysis was carried out as described by [12]. In brief, XOSs and fractions were analysed by TLC using a silica gel 60 plate (Merck KGaA, Darmstadt, Germany). The products were developed with a mixture of 1-butanol, acetic acid, and water in a 2:1:1 (*v*/*v*/*v*) ratio. The products were detected by spraying a mixture of diphenylamine, aniline, acetone, and 80% phosphate in a 2:2:100:15 (*w*/*v*/*v*/*v*) ratio, followed by heating at 100 °C for 10 min using a dry heat block. X1, X2, X3, and DX3 were used as standards.

### 3.5. HPLC Analysis

The distribution and quantification of oligosaccharides in the fractions were determined as previously reported [12]. The analysis was conducted using HPLC equipped with a Sugar-D column (4.6 mm × 250 mm, NACALAI TESQUE, INC., Kyoto, Japan) with a column oven temperature at 40 °C. The products were eluted with an isocratic elution system of acetonitrile/water (4:1, *v*/*v*) at a flow rate of 1.0 mL/min, and the products were detected with a refractive index detector. X1, X2, X3, and DX3 were used as standards.

### 3.6. MGAM Inhibition

For this experiment, recombined human maltase-glucoamylase (MGAM) was prepared as previously reported [38] and dissolved in 0.1 M phosphate buffer (pH 6.4) to produce a solution at 0.5 U/mL. Pre-incubation was initiated by mixing 20 µL of MGAM solution with 20 µL of inhibitor or distilled water for 10 min at 37 °C. Then, 40 µL of 8 mM maltose or phosphate buffer was added and the mixture was incubated at 37 °C for 10 min, and the reaction was stopped by boiling for 10 min and cooling in an ice bath. Glucose was quantified using a Glucose CII-test (Wako Pure Chemical Industries, Osaka, Japan). The 20 µL reaction mixture was added to a 100 µL of Glucose CII-test. The reaction proceeded for 10 min at 37 °C, and the absorbance at 492 nm was measured. The inhibition was analysed at different inhibitor and substrate concentrations. The half-maximum inhibition concentration (IC_50_) was calculated using nonlinear regression. The MGAM inhibitory activity was calculated as follows [39]:

$$\mathrm{MGAM}\;\mathrm{inhibitory}\;\mathrm{activity}=\left(1-\frac{\mathrm{As}-A_{0}}{\mathrm{Ac}}\right)\times 100$$

where As represents the absorbance of the sample, Ac is the absorbance of the control without the inhibitor, and A0 is the absorbance of the sample without MGAM.

### 3.7. α-Amylase Inhibition

The α-amylase from porcine pancreas solution was freshly prepared by mixing enzyme powder with 20 mM phosphate buffer (pH 6.9) containing 6.7 mM NaCl to produce a solution at 3 U/mL [39]. Pre-incubation was initiated by mixing 20 µL of α-amylase solution with 20 µL of inhibitor or distilled water for 10 min at 37 °C. Then, 20 µL of 1% (*w*/*v*) starch (prepared in 20 mM phosphate buffer (pH 6.9) containing 6.7 mM NaCl and heated at 100 °C for 10 min) or phosphate buffer was added, and the mixture was incubated at 37 °C for 10 min. The reaction was stopped by boiling the mixture for 10 min and cooling in an ice bath. The concentration of reducing sugars was calculated by the DNS method [33]. The inhibition was analysed at different inhibitor and substrate concentrations. The half-maximum inhibition concentration (IC_50_) was calculated using nonlinear regression [40]. The α-amylase inhibitory activity was calculated as follows:

$$\alpha -\mathrm{amylase}\;\mathrm{inhibitory}\;\mathrm{activity}=\left(1-\frac{A_{s}-A_{0}}{A_{c}}\right)\times 100$$

where A_s_ represents the absorbance of the sample, A_c_ is the absorbance of the control without the inhibitor, and A_0_ is the absorbance of the sample without α-amylase.

### 3.8. Kinetic Analysis of the Inhibition

To study the kinetics of MGAM and α-amylase inhibition by XOS, the initial velocity in absorbance/min (ΔOD/min) was calculated at different substrate concentrations (0 to 10 mg/mL starch concentration for α-amylase and 0 to 8 mM maltose concentration for MGAM) and XOS concentrations. The values of initial velocity at different substrate concentrations at each XOS concentration was plotted (Michaelis–Menten plot), as well as the double reciprocal plot at each XOS concentration (Lineweaver–Burk plot).

The Michaelis–Menten equation for competitive inhibition is given as follows [41]:

$$v=\frac{V_{\max}\times S}{K_{m}\left(1+\frac{I}{K_{i}}\right)+S}=\frac{V_{\max}^{app}\times S}{K_{m}^{app}+S}$$

And the corresponding Lineweaver–Burk equation is given as follows:

$$\frac{1}{v}=\frac{K_{m}}{V_{\max}}\left(1+\frac{I}{K_{i}}\right)\frac{1}{S}+\frac{1}{V_{\max}}$$

where *v* is the initial velocity, *V*_max_ is the maximum initial reaction velocity, *I* is the concentration of the inhibitor, *S* is the concentration of the substrate, *K*_m_ is the Michaelis constant, and *K*_i_ is the competitive inhibition constant.

### 3.9. Molecular Docking

#### 3.9.1. Ligand Preparation

Acarbose and β-D-Xylopyranose (X1) were retrieved from the PubChem database (ID: 444254 and 125409). X1 was used as template for the construction of the predicted di-, tri-, and tetra-xylooligosaccharides produced from the hydrolysis of xylan. XOSs were built using ChemDraw Pro (CambridgeSoft, Cambridge, MA, USA), and the energy of each molecule was minimized using Discovery Studio Visualizer (https://discover.3ds.com/discovery-studio-visualizer-download accessed on 21 March 2024) in addition to ChemDraw Pro. XOS and Acarbose were then prepared for the flexible docking software using AutoDockTools version 1.5.7.

#### 3.9.2. Protein Preparation

The appropriate three-dimensional structures of human MGAM (PDB ID: 2QMJ) and α-amylase (PDB ID: 1B2Y) were retrieved from the Protein Data Bank (https://www.rcsb.org/ (accessed on 15 January 2022). The human pancreatic α-amylase was used in this study and shares 90% of sequence similarity with the porcine isoform [42]. All the ligands were removed using Biovia Discovery Studio v19.1 software, and the proteins were prepared for docking using AutoDock Tools, and Kollman charges were applied during the preparation process.

#### 3.9.3. Grid Generation and Molecular Docking

The grid was centred on the crystallised ligand in the crystal structure of MGAM and α-amylase using AGFR 1.0 Tool, a dedicated software for flexible docking preparation. The dimensions of the grid box were set as follows: 32 Å × 32 Å × 32 Å, centre-x = −21.066 Å, centre-y = −3.490 Å, and centre-z = 11.440 Å for MGAM, and 18.000 Å × 44.750 Å × 45.000 Å, centre-x = 16.021 Å, centre-y = 10.052 Å, and centre-z = 49.686 Å for α-amylase. Docking was performed at 3 × 10^6^ evaluations in 50 runs each using AutoDockFR software. Results were analysed using PyMOL version 2.5.2, Biovia Discovery Studio, and LigPlot+ version 2.2.5 for the 2D interaction diagram.

## 4. Conclusions

It was assumed in this study that consumption of dulse could exert antidiabetic potential by the prevention of postprandial hyperglycaemia through the inhibition of carbohydrate digestive enzymes, α-amylase and MGAM, by XOSs. Results showed a moderate inhibition by XOS containing a unique β-(1→3) linkage to carbohydrate digestive enzymes, although it was lower than that of acarbose, a reference drug for the treatment of diabetes. The size and molecular structure of XOS affected different effects on the inhibition of α-amylase and MGAM, although the mode of inhibition was competitive. The interactions between individual XOSs with α-amylase and MGAM were also evaluated at the molecular level using molecular docking associated to suitable visualizing tools. In conclusion, the antidiabetic effect of XOSs by preventing postprandial hyperglycaemia could be exerted at usual and continuous consumption as functional foods. Inhibition of blood glucose level is related to the enzymes, not only carbohydrate hydrolase enzymes but also DPP-IV. Revealing the inhibitory effect of XOSs on DPP-IV will provide useful information. Furthermore, this study could find importance in finding new potent XOSs for carbohydrate digestive enzyme inhibition, as new safe functional foods to prevent and treat T2D.

## Figures and Tables

**Table 1:** Kinetics of MGAM inhibition by XOSs from dulse.

Inhibitor (XOS)	*V*_max_^app^ (DO/min)			*K*_m_^app^ (mM)			Inhibition Type	*K*_i_
	A	B	C	A	B	C		(mg/mL)
X2	0.086	0.086	0.086	4.758	5.030	5.403	Competitive	15.20
X3	0.083	0.083	0.083	4.358	4.812	7.646	Competitive	16.00
DX3	0.101	0.093	0.102	4.013	3.743	5.372	Competitive	18.31
DX4	0.102	0.105	0.102	3.940	4.580	5.097	Competitive	23.33

Values are means of duplicate determination. The characters (A, B, and C) represent the concentration of the inhibitor. A–C of X2, 1.49, 2.79, and 3.73 mg/mL; A–C of X3, 1.63, 4.08, and 10.19 mg/mL; A–C of DX3, 1.33, 3.32, and 8.30 mg/mL; A–C of DX4, 1.28, 3.19, and 7.98 mg/mL. In the absence of inhibitor *V*_max_^app^: 0.089 (DO/min) and *K*_m_^app^: 3.197 (mM).

**Table 2:** Kinetics of α-amylase inhibition by XOSs from dulse.

Inhibitor (XOS)	*V*_max_^app^ (DO/min)	*K*_m_^app^ (mg/mL)
X2 (12.42 mg/mL)	0.061	2.064
X3 (13.58 mg/mL)	0.056	1.967

Values are means of duplicate determination. The characters (A and B) represent the concentration of XOSs. A of X2 and X3 are 12.42 mg/mL and 13.58 mg/mL, respectively. In the absence of inhibitor V_max_^app^: 0.056 (DO/min) and K_m_^app^: 0.611 (mg/mL).

**Table 3:** Molecular docking analysis results of oligosaccharides against the structure of MGAM and α-amylase.

Enzyme	Molecule	Pose N°/ Total Poses	Affinity * (kcal/mol)	Number of Hydrogen Bonds	Residues Involved in Hydrogen Bonds	Number of Non-Bonded Contacts
MGAM	Acarbose	1/34	−7.4	13	Tyr299, Asp327, Arg334, Asp443(3) **, Asp542(2), Asp571, Gln603(3), Tyr605	88
	X2	1/7	−5.8	7	Asp327, Trp539, Asp542(3), Asp571, His600	96
	X3	1/9	−5.6	9	Asp203, Thr205(2), Asp327, Trp406, Asp443, Asp542(2), Arg526	63
	DX3	1/21	−5.0	9	Asp203(2), Thr205(2), Tyr214, Asp327, Met444, Arg526, Asp542	64
	DX4	1/32	−6.0	11	Arg202, Asp203(3), Asp327, Trp406, Asp443(2), Ser448, Asp542, Arg526	79
α-amylase	Acarbose	1/9	−5.1	16	Trp59, Arg195, Asp197(2), Glu233(2), His299, Asp300(5), His305(2), Gly306, Asp356	69
	X2	1/4	−5.5	10	Trp59, Tyr62, Arg195, Asp197(2), Glu233(2), His299, Asp300(2)	57
	X3	1/6	−4.9	11	Thr163, Arg195(3), Asp197, Ala198, Glu233, Asp300, His305(2), Gly306	63

*, Affinity energy was calculated by AutoDockFR suite version 1.0 (The Scripps Research Institute, Centre For Computational Structural Biology, La Jolla, CA, USA). The hit with the lower energy was considered. **, Numbers in parenthesis mean the number of hydrogen bonds of the amino acid. Number of hydrogen bonds and non-bonded contact was calculated by LigPlot+ software version 2.2 and Discovery Studio using default parameters.

## Data Availability

Data are contained within the article.

## Supplementary Materials

The following supporting information can be downloaded at https://www.mdpi.com/article/10.3390/molecules29071536/s1, Figure S1: TLC of purified XOSs by activated carbon chromatography; Figure S2: Michaelis–Menten plot for the inhibition of MGAM by XOSs; Figure S3: Michaelis–Menten plot for the inhibition of α-amylase by XOSs; Table S1: Fractionation of XOSs and the yield of fractions, and the purity of given oligosaccharide in the fraction calculated by HPLC analysis.
