# Supplementary material for: Understanding Antidiabetic Potential of Oligosaccharides from Red Alga Dulse Devaleraea inkyuleei Xylan by Investigating α-Amylase and α-Glucosidase Inhibition
Source: Molecules. 2024 Mar 29;29(7):1536. doi: 10.3390/molecules29071536 (PMC11013419; doi:10.3390/molecules29071536)
Supplement: Supplementary file 1 [file molecules-29-01536-s001.zip › molecules-2923601-supplementary.pdf]

## Supplementary Materials

# Understanding Antidiabetic Potential of Oligosaccharides from Red Alga Dulse *Devaleraea inkyuleei* Xylan by Investigating $\alpha$-Amylase and $\alpha$-Glucosidase Inhibition

**Martin Alain Mune Mune [1,*], Tadashi Hatanaka [2], Hideki Kishimura [3], Yuya Kumagai [3,*]**

1 Faculty of Science, University of Maroua, PO Box 814, Maroua, Cameroon; alainmune@gmail.com (M.A.M.M.)
2 Okayama Prefectural Technology Center for Agriculture, Forestry and Fisheries, Research Institute for Biological Sciences (RIBS), Okayama, 7549-1 Kibichuo-cho, Kaga-gun, Okayama, Japan; hatanaka@bio-ribs.com (T.H.)
3 Laboratory of Marine Chemical Resource Development, Faculty of Fisheries Sciences, Hokkaido University, Hakodate, Hokkaido 041-8611, Japan; i-dulse@fish.hokudai.ac.jp (H.K.); yuyakumagai@fish.hokudai.ac.jp (Y.K.)
* Correspondence: alainmune@gmail.com (M.A.M.M.), yuyakumagai@fish.hokudai.ac.jp (Y.K.)

**List of Supplementary Materials**

**Figure S1.** TLC of purified XOS by an activated carbon chromatography.
**Figure S2.** Michaelis-Menten plot for the inhibition of MGAM by XOS.
**Figure S3.** Michaelis-Menten plot for the inhibition of $\alpha$-amylase by XOS.

**Table S1.** Fractionation of XOS and the yield of fractions, and the purity of given oligosaccharide in the fraction calculated by HPLC analysis.

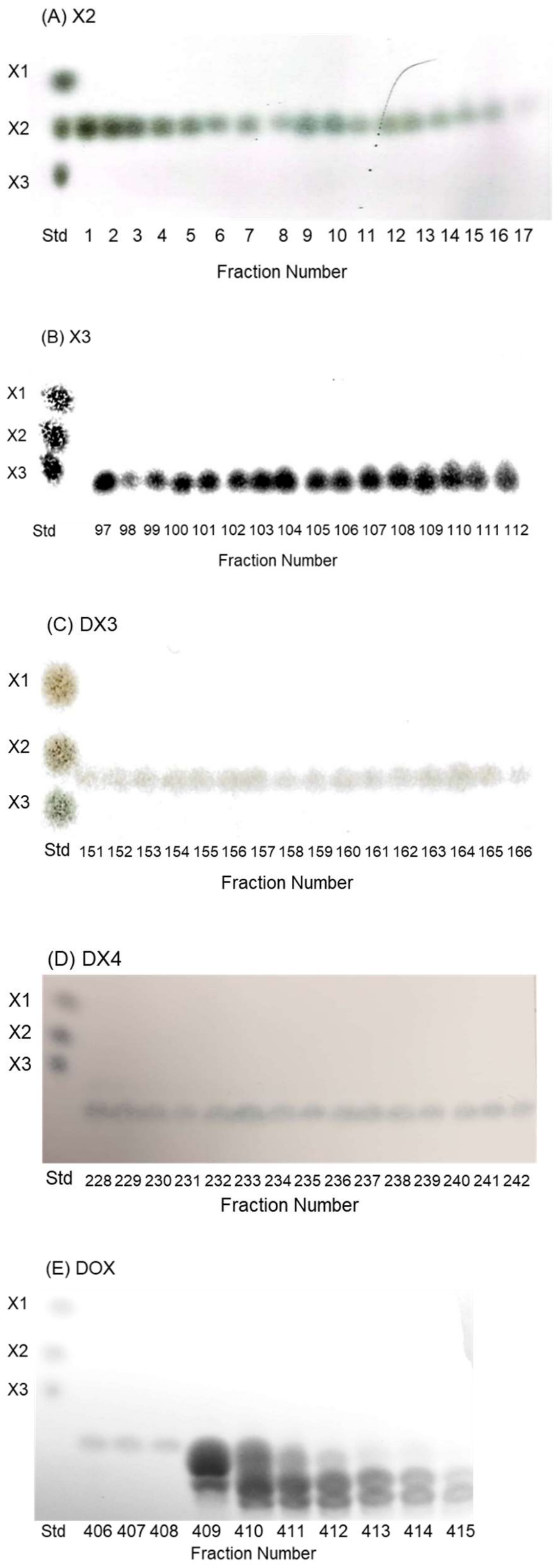


**Figure S1.** TLC of purified XOS by an activated carbon chromatography.

**Table S1.** Fractionation of XOS and the yield of fractions, and the purity of given oligosaccharide in the fraction calculated by HPLC analysis

| Number | Ethanol (%, v/v) | Fraction number | Composition | Mass (g) | Percentage (%) | Yield* (%) | Purity** (%) |
|---|---|---|---|---|---|---|---|
| 1 | 5 | 1-86 | X2 | 0.5416 | 17.59 | 13.54 | 74.5 |
| | 10 | 87-96 | | | | | |
| 2 | 10 | 97-113 | X3 | 0.2193 | 7.12 | 5.48 | 81.5 |
| | 10 | 114-130 | | | | | |
| 3 | 10 | 131-150 | X3-DX3 | 0.0854 | 2.77 | 2.14 | - |
| 4 | 10 | 151-196 | DX3 | 0.1176 | 3.82 | 2.94 | 66.4 |
| | 15 | 197-209 | | | | | |
| 5 | 15 | 210-221 | DX3-DX4 | 0.0572 | 1.86 | 1.43 | - |
| 6 | 15 | 222-308 | DX4 | 0.9056 | 29.49 | 22.64 | 63.8 |
| | 20 | 309-331 | | | | | |
| | 20 | 332-383 | | | | | |
| | 50 | 384-405 | | | | | |
| 7 | 50 | 406-425 | DOX Mixture | 1.1524 | 37.43 | 28.81 | - |
| | | | | | | | |
| Total | | 425 | | 3.0791 | 100 | 76.98 | |

*, Yield = mass of fraction / mass of xylan × 100

**, Purity = mass of XOS / mass of the fraction × 100

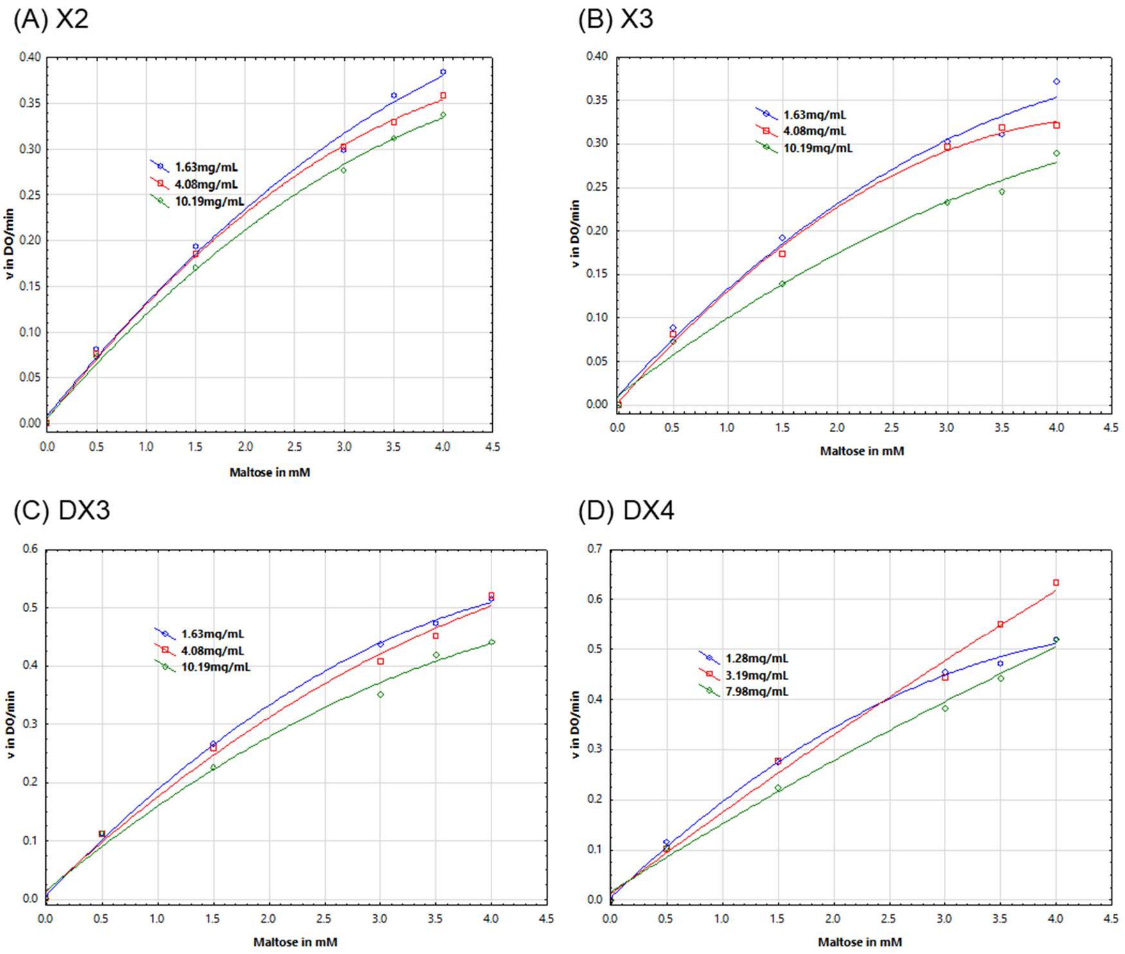


**Figure S2.** Michaelis-Menten plot for the inhibition of MGAM by XOS. (A) X2, (B) X3, (C) DX3, (D) DX4.

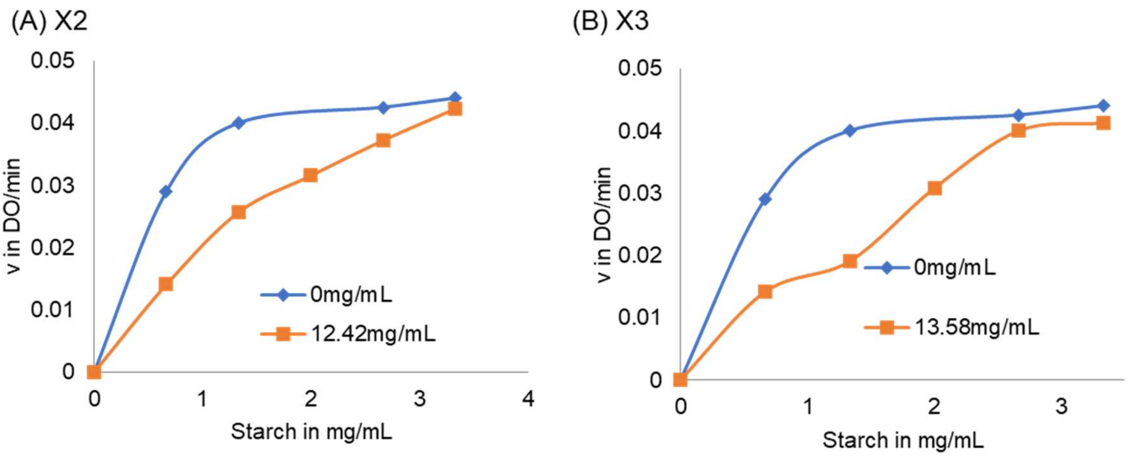


**Figure S3.** Michaelis-Menten plot for the inhibition of $\alpha$-amylase by XOS. (A) X2, (B) X3.
